# 5-Bromo-5-bromo­methyl-2-phen­oxy-1,3,2-dioxaphospho­rinan-2-one

**DOI:** 10.1107/S1600536808033631

**Published:** 2008-10-31

**Authors:** Hoong-Kun Fun, Suchada Chantrapromma, Avijit Kr. Adak, Annada C. Maity, Shyamaprosad Goswami

**Affiliations:** a X-ray Crystallography Unit, School of Physics, Universiti Sains Malaysia, 11800 USM, Penang, Malaysia; b Crystal Materials Research Unit, Department of Chemistry, Faculty of Science, Prince of Songkla University, Hat-Yai, Songkhla 90112, Thailand; c Department of Chemistry, Purdue University, West Lafayette, IN 47907, USA; d Department of Chemistry, Bengal Engineering and Science University, Shibpur, Howrah 711 103, India

## Abstract

In the title 1,3,2-dioxaphospho­rinane derivative, C_10_H_11_Br_2_O_4_P, the 1,3,2-dioxaphospho­rinane ring adopts a chair conformation, having the P=O bond equatorially oriented and the phen­oxy group axially oriented. The bromo substituent is in an axial position opposite to the phen­oxy group and the bromo­methyl group is in an equatorial position opposite to the P=O bond. In the crystal packing, mol­ecules are linked through weak C—H⋯O and C—H⋯Br inter­actions to form chains along the *b* axis. The chains are arranged into sheets parallel to the *ab* plane. In adjacent sheets, mol­ecules are arranged in an anti­parallel fashion. Inter­molecular C—H⋯π inter­actions are also observed.

## Related literature

For values of bond lengths and angles, see: Allen *et al.* (1987 ▶). For hydrogen-bond motifs, see: Bernstein *et al.* (1995 ▶). For ring conformations, see: Cremer & Pople (1975 ▶). For related structures, see, for example: Jones *et al.* (1984 ▶); Polozov *et al.* (1995 ▶). For related literature and applications of dioxaphospho­rinane derivatives, see, for example: Goswami (1993 ▶); Goswami & Adak (2002 ▶); Pilato *et al.* (1991 ▶); Taylor & Goswami (1992 ▶).
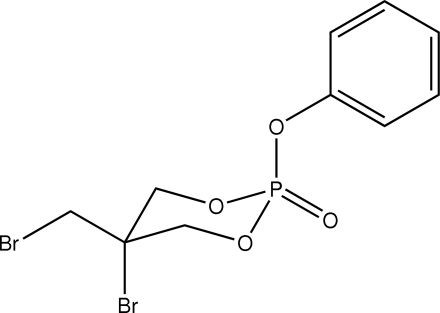


## Experimental

### 

#### Crystal data

C_10_H_11_Br_2_O_4_P
*M* *_r_* = 385.96
Monoclinic, 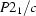

*a* = 12.1315 (3) Å
*b* = 6.3095 (1) Å
*c* = 16.8901 (3) Å
β = 92.196 (2)°
*V* = 1291.88 (4) Å^3^
*Z* = 4
Mo *K*α radiation
μ = 6.40 mm^−1^
*T* = 296 (2) K
0.45 × 0.10 × 0.05 mm

#### Data collection

Bruker APEXII CCD area-detector diffractometer
Absorption correction: multi-scan (*SADABS*; Bruker, 2005 ▶) *T*_min_ = 0.156, *T*_max_ = 0.726
16007 measured reflections
3756 independent reflections
1952 reflections with *I* > 2σ(*I*)
*R*_int_ = 0.072

#### Refinement

*R*[*F* ^2^ > 2σ(*F* ^2^)] = 0.047
*wR*(*F* ^2^) = 0.142
*S* = 0.99
3756 reflections
154 parameters
H-atom parameters constrained
Δρ_max_ = 0.67 e Å^−3^
Δρ_min_ = −0.91 e Å^−3^

### 

Data collection: *APEX2* (Bruker, 2005 ▶); cell refinement: *APEX2*; data reduction: *SAINT* (Bruker, 2005 ▶); program(s) used to solve structure: *SHELXTL* (Sheldrick, 2008 ▶); program(s) used to refine structure: *SHELXTL*; molecular graphics: *SHELXTL*; software used to prepare material for publication: *SHELXTL* and *PLATON* (Spek, 2003 ▶).

## Supplementary Material

Crystal structure: contains datablocks global, I. DOI: 10.1107/S1600536808033631/is2349sup1.cif

Structure factors: contains datablocks I. DOI: 10.1107/S1600536808033631/is2349Isup2.hkl

Additional supplementary materials:  crystallographic information; 3D view; checkCIF report

## Figures and Tables

**Table 1:** Hydrogen-bond geometry (Å, °) *Cg*1 is the centroid of the phenyl ring.

*D*—H⋯*A*	*D*—H	H⋯*A*	*D*⋯*A*	*D*—H⋯*A*
C2—H2*A*⋯O3^i^	0.97	2.35	3.217 (6)	148
C4—H4*A*⋯O3^i^	0.97	2.53	3.362 (6)	144
C2—H2*B*⋯*Cg*1^ii^	0.97	2.81	3.755 (5)	166
C3—H3*C*⋯*Cg*1^iii^	0.97	2.70	3.560 (5)	148

Symmetry codes: (i) 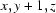; (ii) 
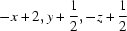
; (iii) 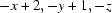.

**Fig. 1.**
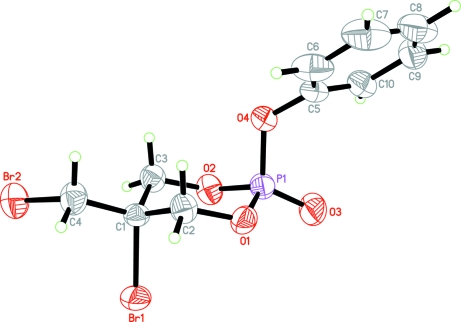
The molecular structure of the title compound, showing 50% probability displacement ellipsoids and the atomic numbering.

**Fig. 2.**
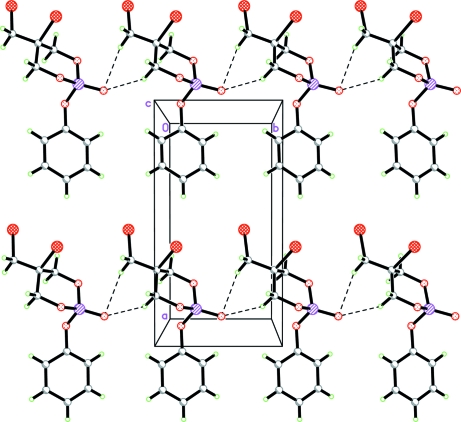
The crystal packing of (I), viewed along the c axis showing chains along the b axis. Hydrogen bonds were shown as dash lines.

**Fig. 3.**
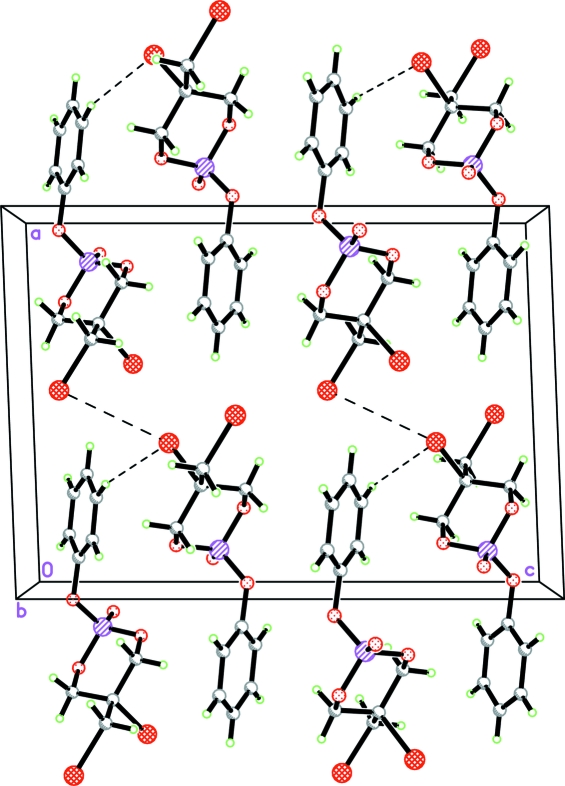
The crystal packing of (I), viewed along the b axis showing the anti-parallel arrangement of the adjacent sheets. Hydrogen bonds and Br···Br short contact were shown as dash lines.

## supplementary crystallographic information

### Comment

Six-membered cyclic phosphates are important constituents present in a number of biologically important molecules *e.g.* cyclic adenosine monophosphate (cAMP) and the Compound *Z*, a precursor of the molybdenum cofactor (Moco) (Goswami, 1993). They especially play key roles in many biosynthetic pathways and comprise structural sub-units of many physiologically important materials. In our synthetic studies (Pilato *et al.*, 1991; Taylor & Goswami, 1992) on the molybdenum cofactor, we are interested to have an efficient synthesis of cyclic dihydroxyacetone phosphate (CDHAP) (Goswami & Adak, 2002). Reaction of phosphate triesters with *N*-bromosuccinimide (NBS) results in the formation of a dibromo derivative (Fig. 1).

In the title 1,3,2-dioxaphosphorinane derivative (Fig. 1), C_10_H_11_Br_2_O_4_P, the 1,3,2-dioxaphosphorinane ring adopts a slightly flattened chair conformation with the puckering parameter (Cremer & Pople, 1975) Q = 0.496 (4) Å, θ = 7.4 (5)° and φ = 177 (4)°, having the P═O bond equatorially attached and the phenoxy substituent axially attached with the torsion angle O1—P1—O4—O5 = 82.6 (4)°. The orientation of the phenoxy group is not co-planar to the 1,3,2-dioxaphosphorinane ring as can be indicated by the torsion angle P1—O4—C5—C6 of -108.2 (4)°. The bromo substituent is in the opposite axial position to the phenoxy substituent and the methylbromo group is in an opposite equatorial position to the P═O bond. The bond lengths and angles in (I) are within normal ranges (Allen *et al.*, 1987) and are comparable to related structures (Jones *et al.*, 1984; Polozov *et al.*, 1995). The closest Br···Br distance is 3.5484 (9) Å.

In the crystal packing shown in Fig. 2, the molecules are linked through weak C—H···O interactions (Table 1) to form chains along the *b* axis which generate *S*(6) ring motifs (Bernstein *et al.*, 1995). The chains are arranged into sheets parallel to the *ab* plane. In the adjacent sheets, the molecules are arranged in an anti-parallel fashion (Fig. 3). The adjacent sheets are connected through weak C—H···O interactions (Table 1) and Br···Br short contacts with the Br···Br distance of 3.8771 (9) Å (symmetry code: 1 - *x*, 1/2 + *y*, 1/2 - *z*). The crystal is stabilized by weak C—H···O, C—H···Br interactions and C—H···π interactions (Table 1); *Cg*1 is the centroid of the C5–C10 ring.

### Experimental

A solution of 5-methylene-2-oxo-2-phenoxy-[1,3,2]-dioxaphosphorinane (0.4 g, 1.76 mmol), doubly crystallized *N*-bromosuccinimide (0.38 g, 1.78 mmol) and azobisisobutyronitrile (10 mg) in dry CCl_4_ (40 ml) was heated under reflux in the presence of a 60 W lamp for 4 h. By this time, a maximum of 80% of the starting materials were converted into the product. Upon prolonged heating for a period of 8 h, no improvement has been observed with respect to yield nor new spot was observed as monitored by thin layer chromatography. The CCl_4_ layer was then stripped off and the gummy material was dissolved in dichloromethane (100 ml) and washed well with water (2 × 100 ml) and then with brine. The organic layer was dried (Na_2_SO_4_) and concentrated to afford the crude product as a light brown gum which was passed through a silica gel (100–200 mesh) column eluting with dichloromethane to get the pure title compound as a white crystalline solid (0.32 g, 60%; m.p. 361–362 K).

### Refinement

All H atoms were constrained in a riding motion approximation, with C_aryl_—H = 0.93 Å and 0.97 Å for CH_2_. The *U*_iso_(H) values were constrained to be 1.2*U*_eq_ of the carrier atom. The highest residual electron density peak is located at 0.72 Å from Br1 and the deepest hole is located at 0.61 Å from Br2.

### Crystal data

**Table:**

C_10_H_11_Br_2_O_4_P	*F*(000) = 752
*M**_r_* = 385.96	*D*_x_ = 1.984 Mg m^−^^3^
Monoclinic, *P*2_1_/*c*	Melting point = 361–362 K
Hall symbol: -P 2ybc	Mo *K*α radiation, λ = 0.71073 Å
*a* = 12.1315 (3) Å	Cell parameters from 3756 reflections
*b* = 6.3095 (1) Å	θ = 2.4–30.0°
*c* = 16.8901 (3) Å	µ = 6.40 mm^−^^1^
β = 92.196 (2)°	*T* = 296 K
*V* = 1291.88 (4) Å^3^	Needle, colourless
*Z* = 4	0.45 × 0.10 × 0.05 mm

### Data collection

**Table:**

Bruker APEXII CCD area-detector diffractometer	3756 independent reflections
Radiation source: fine-focus sealed tube	1952 reflections with *I* > 2σ(*I*)
graphite	*R*_int_ = 0.072
Detector resolution: 8.33 pixels mm^-1^	θ_max_ = 30.0°, θ_min_ = 2.4°
ω scans	*h* = −15→17
Absorption correction: multi-scan (SADABS; Bruker, 2005)	*k* = −8→8
*T*_min_ = 0.156, *T*_max_ = 0.726	*l* = −22→23
16007 measured reflections	

### Refinement

**Table:**

Refinement on *F*^2^	Primary atom site location: structure-invariant direct methods
Least-squares matrix: full	Secondary atom site location: difference Fourier map
*R*[*F*^2^ > 2σ(*F*^2^)] = 0.047	Hydrogen site location: inferred from neighbouring sites
*wR*(*F*^2^) = 0.142	H-atom parameters constrained
*S* = 0.99	*w* = 1/[σ^2^(*F*_o_^2^) + (0.0636*P*)^2^ + 0.3299*P*] where *P* = (*F*_o_^2^ + 2*F*_c_^2^)/3
3756 reflections	(Δ/σ)_max_ = 0.001
154 parameters	Δρ_max_ = 0.67 e Å^−^^3^
0 restraints	Δρ_min_ = −0.91 e Å^−^^3^

### Special details

**Table:**

Geometry. All e.s.d.'s (except the e.s.d. in the dihedral angle between two l.s. planes) are estimated using the full covariance matrix. The cell e.s.d.'s are taken into account individually in the estimation of e.s.d.'s in distances, angles and torsion angles; correlations between e.s.d.'s in cell parameters are only used when they are defined by crystal symmetry. An approximate (isotropic) treatment of cell e.s.d.'s is used for estimating e.s.d.'s involving l.s. planes.
Refinement. Refinement of *F*^2^ against ALL reflections. The weighted *R*-factor *wR* and goodness of fit *S* are based on *F*^2^, conventional *R*-factors *R* are based on *F*, with *F* set to zero for negative *F*^2^. The threshold expression of *F*^2^ > σ(*F*^2^) is used only for calculating *R*-factors(gt) *etc*. and is not relevant to the choice of reflections for refinement. *R*-factors based on *F*^2^ are statistically about twice as large as those based on *F*, and *R*- factors based on ALL data will be even larger.

### Fractional atomic coordinates and isotropic or equivalent isotropic displacement parameters (Å^2^)

**Table:**

	*x*	*y*	*z*	*U*_iso_*/*U*_eq_	
Br1	0.60424 (5)	0.36096 (9)	0.21315 (3)	0.0610 (2)	
Br2	0.53167 (5)	0.78769 (10)	0.08461 (4)	0.0719 (2)	
P1	0.88523 (11)	0.20179 (19)	0.12969 (8)	0.0438 (3)	
O1	0.8686 (3)	0.3316 (5)	0.20749 (18)	0.0463 (8)	
O2	0.7744 (3)	0.2261 (5)	0.0802 (2)	0.0495 (8)	
O3	0.9159 (3)	−0.0157 (5)	0.1448 (3)	0.0677 (11)	
O4	0.9665 (3)	0.3340 (5)	0.0784 (2)	0.0504 (8)	
C1	0.7093 (4)	0.5327 (6)	0.1542 (3)	0.0376 (10)	
C2	0.8186 (4)	0.5375 (7)	0.2016 (3)	0.0443 (11)	
H2A	0.8687	0.6340	0.1764	0.053*	
H2B	0.8063	0.5908	0.2544	0.053*	
C3	0.7229 (4)	0.4330 (7)	0.0740 (3)	0.0453 (11)	
H3B	0.6511	0.4192	0.0472	0.054*	
H3C	0.7677	0.5250	0.0422	0.054*	
C4	0.6653 (4)	0.7573 (7)	0.1467 (3)	0.0519 (13)	
H4A	0.7214	0.8449	0.1235	0.062*	
H4B	0.6535	0.8115	0.1994	0.062*	
C5	1.0807 (4)	0.3235 (7)	0.0921 (3)	0.0408 (11)	
C6	1.1335 (5)	0.4950 (9)	0.1258 (3)	0.0619 (15)	
H6A	1.0939	0.6132	0.1413	0.074*	
C7	1.2469 (6)	0.4884 (12)	0.1362 (3)	0.077 (2)	
H7A	1.2841	0.6040	0.1585	0.092*	
C8	1.3055 (5)	0.3123 (14)	0.1139 (3)	0.077 (2)	
H8A	1.3817	0.3087	0.1217	0.092*	
C9	1.2510 (5)	0.1428 (10)	0.0803 (3)	0.0640 (16)	
H9A	1.2904	0.0241	0.0651	0.077*	
C10	1.1368 (4)	0.1479 (8)	0.0688 (3)	0.0494 (12)	
H10A	1.0993	0.0336	0.0458	0.059*	

### Atomic displacement parameters (Å^2^)

**Table:**

	*U*^11^	*U*^22^	*U*^33^	*U*^12^	*U*^13^	*U*^23^
Br1	0.0439 (3)	0.0719 (4)	0.0680 (4)	−0.0032 (3)	0.0128 (3)	0.0258 (3)
Br2	0.0560 (4)	0.0786 (4)	0.0810 (5)	0.0177 (3)	0.0001 (3)	0.0093 (3)
P1	0.0372 (7)	0.0360 (6)	0.0585 (8)	−0.0025 (5)	0.0049 (6)	0.0049 (5)
O1	0.0419 (19)	0.0494 (18)	0.0472 (19)	0.0009 (15)	−0.0047 (15)	0.0109 (14)
O2	0.048 (2)	0.0423 (17)	0.058 (2)	0.0006 (15)	−0.0007 (17)	−0.0115 (15)
O3	0.056 (2)	0.0374 (18)	0.110 (3)	0.0033 (17)	0.011 (2)	0.0135 (19)
O4	0.0383 (19)	0.0516 (19)	0.062 (2)	0.0020 (15)	0.0096 (16)	0.0124 (16)
C1	0.038 (3)	0.035 (2)	0.040 (2)	−0.0041 (19)	0.006 (2)	0.0020 (18)
C2	0.041 (3)	0.047 (3)	0.044 (3)	0.001 (2)	−0.003 (2)	−0.004 (2)
C3	0.040 (3)	0.052 (3)	0.043 (3)	0.001 (2)	−0.005 (2)	0.000 (2)
C4	0.044 (3)	0.041 (3)	0.070 (4)	−0.001 (2)	−0.002 (3)	−0.001 (2)
C5	0.039 (3)	0.046 (3)	0.037 (2)	−0.007 (2)	0.008 (2)	0.0027 (19)
C6	0.076 (4)	0.056 (3)	0.055 (3)	−0.022 (3)	0.020 (3)	−0.009 (3)
C7	0.082 (5)	0.097 (5)	0.050 (3)	−0.048 (4)	−0.001 (3)	−0.007 (3)
C8	0.048 (4)	0.137 (6)	0.045 (3)	−0.029 (4)	−0.008 (3)	0.022 (4)
C9	0.049 (3)	0.088 (4)	0.055 (3)	0.010 (3)	0.004 (3)	0.014 (3)
C10	0.043 (3)	0.058 (3)	0.048 (3)	−0.005 (2)	0.004 (2)	−0.003 (2)

### Geometric parameters (Å, °)

**Table:**

Br1—C1	1.971 (4)	C3—H3C	0.9700
Br2—C4	1.907 (5)	C4—H4A	0.9700
P1—O3	1.442 (4)	C4—H4B	0.9700
P1—O2	1.563 (4)	C5—C10	1.365 (7)
P1—O1	1.568 (3)	C5—C6	1.371 (7)
P1—O4	1.576 (3)	C6—C7	1.380 (9)
O1—C2	1.436 (5)	C6—H6A	0.9300
O2—C3	1.450 (6)	C7—C8	1.380 (10)
O4—C5	1.398 (6)	C7—H7A	0.9300
C1—C3	1.509 (6)	C8—C9	1.369 (9)
C1—C4	1.518 (6)	C8—H8A	0.9300
C1—C2	1.523 (6)	C9—C10	1.392 (8)
C2—H2A	0.9700	C9—H9A	0.9300
C2—H2B	0.9700	C10—H10A	0.9300
C3—H3B	0.9700		
			
O3—P1—O2	113.5 (2)	H3B—C3—H3C	107.9
O3—P1—O1	112.9 (2)	C1—C4—Br2	115.4 (3)
O2—P1—O1	105.14 (18)	C1—C4—H4A	108.4
O3—P1—O4	116.0 (2)	Br2—C4—H4A	108.4
O2—P1—O4	101.35 (19)	C1—C4—H4B	108.4
O1—P1—O4	106.73 (19)	Br2—C4—H4B	108.4
C2—O1—P1	118.8 (3)	H4A—C4—H4B	107.5
C3—O2—P1	119.1 (3)	C10—C5—C6	122.0 (5)
C5—O4—P1	121.4 (3)	C10—C5—O4	119.5 (4)
C3—C1—C4	111.3 (4)	C6—C5—O4	118.4 (5)
C3—C1—C2	110.9 (4)	C5—C6—C7	118.5 (6)
C4—C1—C2	108.8 (4)	C5—C6—H6A	120.8
C3—C1—Br1	108.6 (3)	C7—C6—H6A	120.8
C4—C1—Br1	108.9 (3)	C8—C7—C6	120.7 (6)
C2—C1—Br1	108.2 (3)	C8—C7—H7A	119.6
O1—C2—C1	112.0 (4)	C6—C7—H7A	119.6
O1—C2—H2A	109.2	C9—C8—C7	119.8 (6)
C1—C2—H2A	109.2	C9—C8—H8A	120.1
O1—C2—H2B	109.2	C7—C8—H8A	120.1
C1—C2—H2B	109.2	C8—C9—C10	120.1 (6)
H2A—C2—H2B	107.9	C8—C9—H9A	120.0
O2—C3—C1	111.8 (4)	C10—C9—H9A	120.0
O2—C3—H3B	109.3	C5—C10—C9	118.9 (5)
C1—C3—H3B	109.3	C5—C10—H10A	120.5
O2—C3—H3C	109.3	C9—C10—H10A	120.5
C1—C3—H3C	109.3		
			
O3—P1—O1—C2	−168.7 (3)	C2—C1—C3—O2	53.4 (5)
O2—P1—O1—C2	−44.4 (4)	Br1—C1—C3—O2	−65.5 (4)
O4—P1—O1—C2	62.7 (4)	C3—C1—C4—Br2	54.6 (5)
O3—P1—O2—C3	168.0 (3)	C2—C1—C4—Br2	177.1 (3)
O1—P1—O2—C3	44.1 (4)	Br1—C1—C4—Br2	−65.2 (4)
O4—P1—O2—C3	−66.9 (4)	P1—O4—C5—C10	74.5 (5)
O3—P1—O4—C5	−44.2 (4)	P1—O4—C5—C6	−108.2 (4)
O2—P1—O4—C5	−167.6 (3)	C10—C5—C6—C7	0.0 (8)
O1—P1—O4—C5	82.6 (4)	O4—C5—C6—C7	−177.3 (4)
P1—O1—C2—C1	52.5 (5)	C5—C6—C7—C8	−0.6 (9)
C3—C1—C2—O1	−54.0 (5)	C6—C7—C8—C9	0.7 (9)
C4—C1—C2—O1	−176.7 (4)	C7—C8—C9—C10	−0.2 (9)
Br1—C1—C2—O1	65.1 (4)	C6—C5—C10—C9	0.4 (7)
P1—O2—C3—C1	−52.0 (5)	O4—C5—C10—C9	177.7 (4)
C4—C1—C3—O2	174.6 (4)	C8—C9—C10—C5	−0.4 (8)

### Hydrogen-bond geometry (Å, °)

**Table:**

*D*—H···*A*	*D*—H	H···*A*	*D*···*A*	*D*—H···*A*
C2—H2A···O3^i^	0.97	2.35	3.217 (6)	148
C3—H3B···Br2	0.97	2.82	3.233 (5)	106
C4—H4A···O3^i^	0.97	2.53	3.362 (6)	144
C2—H2B···Cg1^ii^	0.97	2.81	3.755 (5)	166
C3—H3C···Cg1^iii^	0.97	2.70	3.560 (5)	148

Symmetry codes: (i) *x*, *y*+1, *z*;  (ii) −*x*+2, *y*+1/2, −*z*+1/2; (iii) −*x*+2, −*y*+1, −*z*.
